# Optimizing 3D Printed Metallic Object’s Postprocessing: A Case of Gamma-TiAl Alloys

**DOI:** 10.3390/ma14051246

**Published:** 2021-03-05

**Authors:** M. A. K. Chowdhury, AMM Sharif Ullah, Roberto Teti

**Affiliations:** 1 Faculty of Science and Technology, Federation University, Mt Helen, VIC 3350, Australia; aa.chowdhury@federation.edu.au; 2 Division of Mechanical and Electrical Engineering, Faculty of Engineering, Kitami Institute of Technology, 165 Koen-cho, Kitami 090-8507, Japan; 3 Department of Chemical, Materials and Industrial, Production Engineering, University of Naples Federico II, Piazzale Tecchio 80, I-80125 Naples, Italy; roberto.teti@unina.it

**Keywords:** electron beam melting (EBM), γ-TiAl, turning, surface roughness, cutting force, 3D printing, postprocessing

## Abstract

Gamma-TiAl (γ-TiAl) alloys can be used in high-end products relevant to the aerospace, defense, biomedical, and marine industries. Fabricating objects made of γ-TiAl alloys needs an additive manufacturing process called Electron Beam Melting (EBM) or other similar processes because these alloys are difficult-to-cut materials. An object fabricated by EBM exhibits poor surface finish and must undergo postprocessing. In this study, cylindrical specimens were fabricated by EBM and post-processed by turning at different cutting conditions (cutting speed, depth of cut, feed rate, insert radius, and coolant flowrate). The EBM conditions were as follows: average powder size 110 μm, acceleration voltage 60 kV, beam current 10 mA, beam scanning speed 2200 mm/s, and beam focus offset 0.20 mm. The surface roughness and cutting force were recorded for each set of cutting conditions. The values of the cutting conditions were set by the L36 Design of Experiment approach. The effects of the cutting conditions on surface roughness and cutting force are elucidated by constructing the possibility distributions (triangular fuzzy numbers) from the experimental data. Finally, the optimal cutting conditions to improve the surface finish of specimens made of γ-TiAl alloys are determined using the possibility distributions. Thus, this study’s outcomes can be used to develop intelligent systems for optimizing additive manufacturing processes.

## 1. Introduction

Gamma-TiAl (γ-TiAl) can replace titanium alloys and Inconel-718 in many engineering applications relevant to the aerospace, marine, and biomedical industry [1] because these alloys exhibit superior material properties such as high creep resistance, oxidation, and burn resistance, enhanced strength at elevated temperatures, and considerably higher specific modulus and lower density [2]. These material properties are desirable when tough environmental legislations are imposed to reduce carbon emission and fuel consumption, which is the case now. In addition, γ-TiAl alloys are biocompatible and can be used in implants replacing Ti6Al4V [3,4].

Significant efforts have been made to understand the manufacturability of γ-TiAl alloys. Still, these alloys are difficult-to-manufacture materials. For example, consider the findings in Harding et al. [5]. In this study, it was found that γ-TiAl alloys are not suitable for forging and other deformation-based processes due to poor ductility and low fracture toughness. For this reason, most of the components are manufactured using casting. TiAl alloy casting has several limitations, such as internal cavity formation, residual stress development, and limitations on shape complexity [6].

Recently, additive manufacturing or 3D printing [7] such as Electron Beam Melting (EBM) has been considered a useful technology to produce parts from titanium and nickel-based superalloys [6,7,8,9,10,11,12]. EBM selectively melts and solidifies the metal powder layer by layer according to the CAD data of an object. A highly focused and energized electron beam is used for melting the powder. Parts produced from EBM have several advantages as compared to conventional casting, such as the following:

1. more complex and unique geometries (internal channels and cavities) can be produced by EBM [13],
2. the cost of expensive tooling (dies and molds) can be eliminated,
3. oxidation- and impurity-free parts can be achieved since the process is accomplished in a vacuum [12], and
4. uniform microstructures can be maintained while printing the part [14].

Furthermore, by this time, some research studies have focused on additive manufacturing’s environmental impacts to make this technology more sustainable [15,16]. However, despite the abovementioned advantages, the typical surface roughness of EBM-manufactured parts belongs to the range 15.8 µm to 54.3 µm [17,18], which is considerably higher than that of the traditional manufacturing processes [19]. This limits the usages of EMB-manufactured parts [20]. For example, Karlsson et al. [21] found that the poor surface finish remains a bottleneck for additively manufactured parts. This is especially severe for smaller parts (<1 cm), where the accuracy of the highly detailed features is lost due to the powder particle size and severe conditions required for melting [13]. Kumar et al. [22] produced EBM parts using the powder of TiAl and developed the relationships between the process parameters and resulting microstructures. Biamino et al. [12] and Mohammad et al. [23] examined the effects of the EBM input parameters on the mechanical properties and the resulting microstructures in γ-TiAl (Ti-48Al-2Cr-2Nb) parts. They concluded that the EBM-produced parts made of γ-TiAl had low porosity, high strength, homogeneous microstructure, and an insignificant level of oxygen-based impurities. Mohammad et al. [24] used a central composite design approach for optimizing the EBM input parameters to maximize the density and improve the surface finish of the additively produced γ-TiAl parts. According to this study, the minimum surface finish is about 5 µm on the top face along the build direction and 25 µm on the side surfaces. These roughness values are significantly higher than those produced by conventional machining processes [25]. Thus, postprocessing is necessary to improve the surface finished of EBM-produced parts [20].

From the machining point of view, γ-TiAl is considered a very difficult-to-cut material due to its high strength, poor thermal conductivity, high chemical affinity with oxygen and cutting tool materials, and its tendency to cause rapid tool wear [26,27]. A notable feature of the γ-TiAl alloys is that their properties are mainly microstructure-dependent, which is strongly influenced by the history of the process route and the heat treatment. It has been reported that the machining of EBM-produced parts is more difficult compared to their wrought counterparts due to the excessive strength and hardness [28,29]. However, the EBM components machining is necessary to improve the surface finish. A few articles have investigated the machinability of the additively manufactured γ-TiAl alloys. For example, the machinability of EBM-produced gamma titanium aluminide alloy was investigated in [30]. They performed milling operations and studied the effects of cutting speed, feed, and cutting fluid conditions (wet, dry, and minimum quantity lubrication (MQL)) on the tool life, surface roughness, and chip morphology. Priarone et al. [31] reported that high tool wear during traditional milling operations of the EBM-produced γ-TiAl makes its machining difficult. The machinability could be improved by applying nano-structured coatings and adjusting the tool geometry.

A comparative study of the flood, dry, and MQL cooling was presented in [32] while turning a specimen EBM-produced γ-TiAl. It was concluded that the flood cooling resulted in minimal tool wear. In [33], an integrated post-processing strategy combining longitudinal milling with fine abrasive finishing is presented to enhance 3D printed parts’ surface integrity. This strategy could produce a surface finish up to 25 nm and decrease the surface porosity by 89%. The study reported in [34] utilized rotary ultrasonic machining (RUM) to improve the surface finish of EBM-manufactured titanium alloy (Ti-6Al-4V). After detailed experimentation based on the design of experiments, an optimized set of RUM parameters was recommended to reach a minimum surface roughness of Ra 0.3 µm. However, the RUM is an expensive process because it needs cutting tools with diamond abrasives. Studies have also reported applying laser ablation to improve the surface finish of EBM-produced parts [18] and selective laser-melted parts [35,36]. However, thermal processing such as laser machining results in recast layers and a heat-affected zone, resulting in a poor surface finish [37].

In this article, EBM is used to produce specimens made of γ-TiAl under the optimal process parameters. Moreover, the specimens are turned as a part of preprocessing to improve their surface finish. Finally, the optimal conditions are identified by quantifying the uncertainty using possibility distributions [38]. It is worth mentioning that there is no study reporting a systematic analysis on how to elucidate the relationships among the conditions (cutting speed, depth of cut, feed rate, insert radius, and coolant flow rate) and cutting performances (cutting force and surface roughness) while turning a 3D-printed object made of γ-TiAl alloys. This study fills this gap. However, one of the limitations of this study is that it does not consider the environmental aspect of EBM. The rest of this article is organized as follows. Section 2 describes the specimen perpetration process and the setting of the design of the experiment for postprocessing. Section 3 describes the data analysis and optimization using the possibility distribution concept, quantifying the uncertainty of the turning conditions and process performance (surface finish and cutting force). Section 4 concludes this article.

## 2. Experimentation

**Figure 1:**
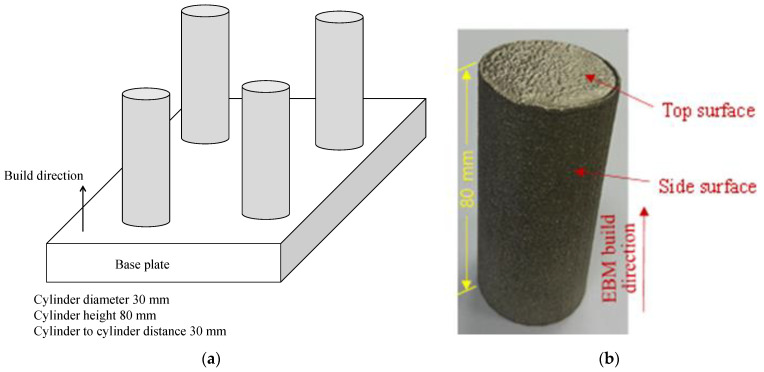
(**a**) Schematics of the parts layout for Electron Beam Melting (EBM) operations, (**b**) actual γ-TiAl cylinder fabricated by EBM.

Solid cylinders (see Figure 1) of 30 mm diameter and 80 mm length were fabricated using EBM (Arcam Q10 Plus technology, GE Additive, Munich, Germany). The γ-TiAl powder with a nominal composition of Ti-46Al-2Cr-2Nb (at.%) was used. The powder particles are spherical with a diameter was in the range of 50 μm to 120 μm. The solid cylinders were built on the baseplate, a 10 mm thick square plate (100 mm by 100 mm) made of stainless steel. Four cylinders were placed at 30 mm from each other, which helped avoid thermal shadowing. Each cylinder’s bottom edge was set 5 mm from the baseplate’s edge to ensure the right melting during EBM. The processing parameters of EBM, such as Beam current, Voltage, Beam scan speed, and Beam focus offset, were set according to values shown in Table 1. The values of the EBM parameters were set based on the studies of Mohammad et al. [23,24]. It can be mentioned here that before spreading the first layer of γ-TiAl powder, the baseplate was pre-heated at 1050 °C.

**Figure 2:**
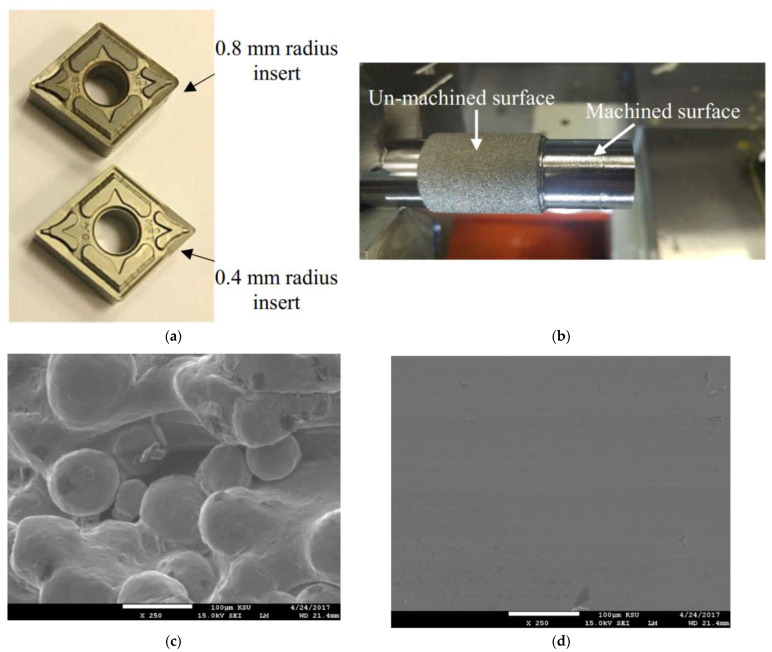
(**a**) Tungsten carbide cutting inserts for turning, (**b**) clamped γ-TiAl cylinder on the chuck during turning experimentation, (**c**) SEM photograph of un-machined EBM produced γ-TiAl cylinder, (**d**) SEM photograph of machined EBM produced γ-TiAl cylinder.

Table 1 and Table 2 list the EBM conditions and physical properties of γ-TiAl alloys. As seen in Figure 1, the fabricated specimens exhibit poor surface texture. A device called Surtronic S100 (Taylor Hobson, Leicester, UK) was used to measure the surface roughness. The surface roughness in terms of the arithmetic average height (*Ra*) of the specimens was about 35 µm and 8 µm on the circumferential and top surfaces, respectively. Such a high surface roughness limits the usages of EBM-produced parts, as mentioned in Section 1. The specimens were postprocessed by performing turning, which improved the surface roughness (see the next section for the results). A machining center (OKUMA LU3000EX, Okumar America Limited, Charlotte, NC USA) performed the turning operations. Two types of uncoated negative-raked tungsten carbide inserts (denoted as CNMG-120404 and CNMG-120408) were used. One of the tools had a nose radius of 0.4 mm, and the other hada nose radius of 0.8 mm (see Figure 2a). A water-miscible oil coolant (Fuchs Ecocool S-HL, FUCHS Group, Jeddah, KSA) was used. The coolant was applied through a flood coolant nozzle.

A set of turning experiments was conducted varying the cutting speed (*v_c_*), depth of cut (*a_p_*), feed rate (*f*), and coolant flowrate (*Q*). The levels of the conditions are shown in Table 3. The levels are like those of other authors [26,39]. In this study, a preliminary turning of the γ-TiAl cylinders was performed to eliminate the non-uniformity that appeared on the surfaces of the cylinders using *a_p_* = 0.1 mm, *f* = 0.075 mm/rev, and *V* = 40 m/min before actual machining experiments.

A total of 36 experiments were conducted based on Taguchi’s L36 experimental design, as listed in Table 4. Pictures of the experimental settings and outcomes are shown in Figure 2. Each time, the cutting force and the surface roughness were measured. The tungsten carbide inserts used are shown in Figure 2a. The surfaces and microstructures after machining and before machining are shown in Figure 2b–d. A dynamometer (Kistler 9257 with charge amplifier type 5070) was used to measure the cutting force. Table 5 lists the cutting force datasets against the respective cutting conditions. The surface roughness of the machined parts was quantified using two parameters, namely the arithmetic average height and the total height of the roughness profile denoted as *R_a_* and *R_t_*. For each machined part, the roughness readings were taken at three random locations of each specimen. The datasets obtained are listed in Table 6 and Table 7. Table 6 lists the datasets of the arithmetic average height of roughness profiles. On the other hand, Table 7 lists the datasets of the total height of roughness profiles. The optimization of cutting force and surface roughness is presented in the next section.

## 3. Optimization

The datasets listed in Table 5, Table 6 and Table 7 were used to identify the optimal cutting conditions. In this respect, a possibility distribution-based method was used because it effectively quantifies the uncertainty associated with the design of experiment datasets [38]. The method induces a possibility distribution (a fuzzy number) for a given dataset using the probability–possibility transformation [42]. Optimization can be achieved by comparing two or more possibility distributions in the same universe of discourse of a process performance parameter. For this particular case, the possibility distributions (in this case, all triangular fuzzy numbers) are induced using the datasets listed in Table 5, Table 6 and Table 7, as described in [38]. The distributions show the effects of the cutting conditions on the respective performance parameters (cutting force and surface roughness). The description is as follows. 

**Figure 3:**
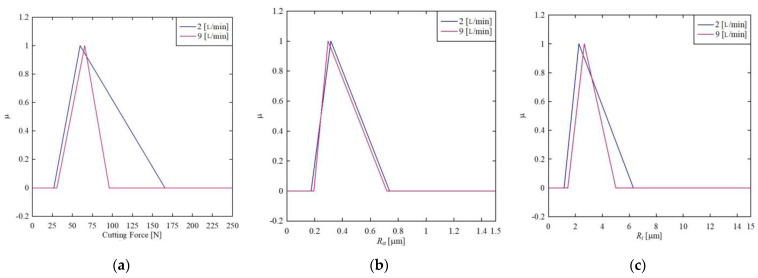
Effect of coolant flowrate on (**a**) cutting force (**b**) *R_a_* (**c**) *R_t_.*

First, consider the effects of coolant flowrate, *Q*, (L/min) on the cutting force, *R_a_*, and *R_t_*. The triangular fuzzy numbers induced are shown in Figure 3. As seen in Figure 3a, when the coolant flowrate is 9 L/min, it minimizes cutting force, whereas the cutting force varies greatly when the coolant flowrate is reduced to 2 L/min. Therefore, keeping coolant flowrate high is a better option to minimize cutting force. On the other hand, surface roughness in terms of *R_a_* cannot be controlled by controlling the coolant flowrate, as seen in Figure 3b. As a result, the coolant flowrate is not effective in minimizing or maximizing *R_a_*. The same argument is somewhat true for *R_t_*, as shown in Figure 3c. However, a high coolant flowrate can keep the surface roughness in terms of *R_t_* to a stipulated range more tightly (compare Figure 3b–c). The expected values of cutting force and surface roughness are calculated using the centroid method for quantitative analysis. The results are as follows, which reconfirm the above conclusions. When the flow rate is increased to 9 L/min from 2 L/min, the expected cutting force based on the centroid method decreases to 64 N from 84 N. When the flow rate is increased to 9 L/min from 2 L/min, the expected value of the surface roughness (*R_a_*) decreases to 0.4027 μm from 0.409 μm. When the flow rate is increased to 9 L/min from 2 L/min, the expected value of the surface roughness (*R_t_*) increases to 3.24 μm from 3.033 μm.

**Figure 4:**
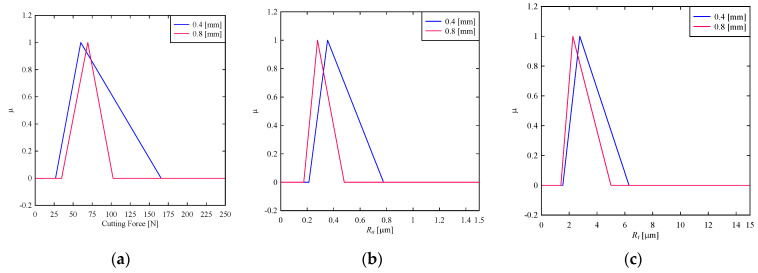
Effect of insert’s nose radius on (**a**) cutting force (**b**) *R_a_* (**c**) *R_t_.*

Secondly, consider the effect of the insert’s nose radius, *r_ε_*, (mm) on the cutting force and surface roughness. As seen in Figure 4a, the cutting force can only be kept to a certain range if the nose radius is 0.8 mm. This means that a large tool nose radius better controls the cutting force and minimizes it (cutting force). As seen in Figure 4b, surface roughness in terms of *R_a_* can be minimized, keeping the nose radius to 0.8 mm. This means that maximizing nose radius minimizes *R_a_*. The same argument is true for *R_t_*, as seen in Figure 4c. Like the previous parameter, the expected values of cutting force and surface roughness are calculated using the centroid method. The results are presented as follows, which confirm the abovementioned qualitative analysis. When the nose radius is increased to 0.8 mm from 0.4 mm, the expected cutting force decreases to 68.8 N from 84.13 N. When the nose radius is increased to 0.8 mm from 0.4 mm, the expected surface roughness (*R_a_*) decreases to 0.311 μm from 0.448 μm. When the nose radius is increased to 0.8 mm from 0.4 mm, the expected value of the surface roughness (*R_t_*) decreases to 2.887 μm from 3.53 μm.

**Figure 5:**
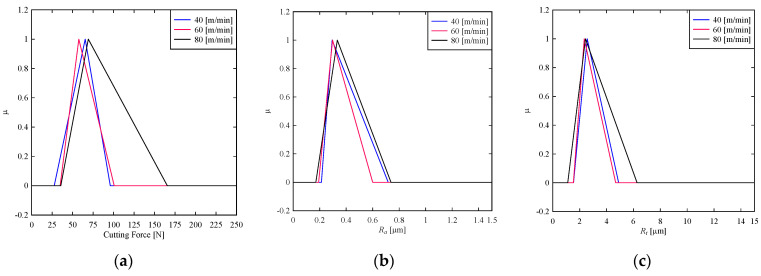
Effect of cutting speed on (**a**) cutting force (**b**) *R_a_* (**c**) *R_t_.*

Thirdly, consider the effect of cutting speed, *v_c_*, (m/min). As shown in Figure 5a, both cutting speeds of 40 m/min and 60 m/min minimize cutting force. This means that a cutting speed up to 60 m/min ensures low cutting force. This is not the case for the other cutting speed (80 m/min). As seen in Figure 5b, the surface roughness in terms of *R_a_* can be minimized, keeping the cutting speed to 60 m/min. However, the other two cutting speeds also ensure almost the same *R_a_*. This means that *R_a_* does not depend much on the cutting speed. The same argument is true for *R_t_*, as shown in Figure 5c. Apart from the abovementioned qualitative results, the expected values of the cutting force and surface roughness are calculated using the centroid method for quantitative analysis. The results are shown as follows, which is consistent with the abovementioned qualitative analysis. The expected values of cutting force are 63 N, 64.4 N, and 90.13 N for cutting speeds 40 m/min, 60 m/min, and 80 m/min, respectively. The expected values of surface roughness in terms of *R_a_* are 0.408 μm, 0.363 μm, and 0.414 μm for cutting speeds 40 m/min, 60 m/min, and 80 m/min, respectively. The expected values of surface roughness in terms of *R_t_* are 3 μm, 2.85 μm, and 3.27 μm for cutting speeds 40 m/min, 60 m/min, and 80 m/min, respectively.

**Figure 6:**
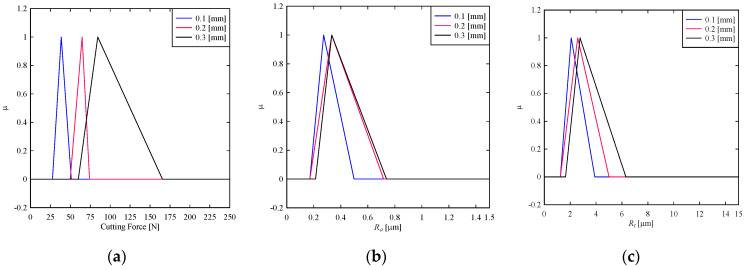
Effect of depth of cut on (**a**) cutting force (**b**) *R_a_* (**c**) *R_t_.*

As seen in Figure 6a, the cutting force decreases with the reduction of the depth of cut. This means that minimizing the cutting force requires minimization of the depth of cut. As seen in Figure 6b, the depth of cut of 0.1 mm ensures a low cutting force. This is not the case for the other two depth of cuts, 0.2 mm and 0.3 mm. As seen in Figure 6c, though the depth of cut of 0.1 mm ensures slightly lower *R_a_*, the other two do not lower *R_a_*. The same argument holds for *R_t_*. For quantitative analysis, the expected values of the cutting force and surface roughness are calculated using the centroid method. The results are shown as follows, which reconfirm the abovementioned qualitative analysis. The expected values of cutting force are 39.13 N, 62.9 N, and 103.33 N for depth of cuts 0.1 m, 0.2 mm, and 0.3 mm, respectively. The expected values of surface roughness in terms of *R_a_* are 0.314 μm, 0.407 μm, and 0.428 μm for the depth of cuts 0.1 mm, 0.2 mm, and 0.3 mm, respectively. The expected values of surface roughness in terms of *R_t_* are 2.407 μm, 2.94 μm, and 3.57 μm for depth of cuts 0.1 mm, 0.2 mm, and 0.3 mm, respectively.

**Figure 7:**
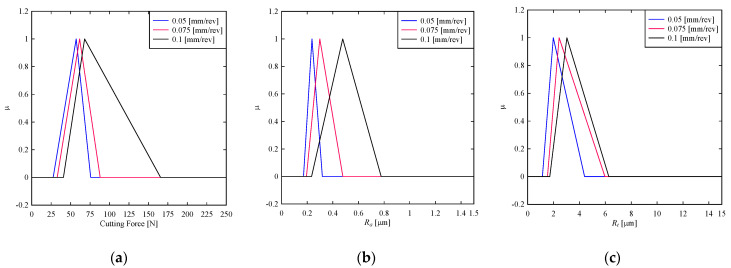
Effect of feed rate on (**a**) cutting force (**b**) *R_a_* (**c**) *R_t_.*

Lastly, consider the effects of feed rate, *f*, (mm/rev), on the cutting force and surface roughness, as shown in Figure 7. The feed rates up to 0.075 mm/rev can minimize the cutting force, and the feed rate of 0.05 mm/rev exhibits the lowest cutting force, as shown in Figure 7a. The uncertainty increases when the feed rate is 0.1 mm/rev. Thus, the feed rate of 0.05 mm/rev is recommended for minimizing cutting force. A similar argument holds for surface roughness. As seen in Figure 7b, minimizing the feed rate minimizes the surface roughness in terms of *R_a_*. The same argument is true for *R_t_*, as seen in Figure 7c. For quantitative analysis, the expected values of the cutting force and surface roughness are calculated using the centroid method. The results are shown as follows, which reconfirm the abovementioned qualitative analysis. The expected values of cutting force are 53.53 N, 60.8 N, and 91.467 N for feed rates 0.05 mm/rev, 0.075 mm/rev, and 0.1 mm/rev, respectively. The expected values of surface roughness in terms of *R_a_* are 0.242 μm, 0.323 μm, and 0.496 μm for feed rates 0.05 mm/rev, 0.075 mm/rev, and 0.1 mm/rev, respectively. The expected values of surface roughness in terms of *R_t_* are 2.51 μm, 3.31 μm, and 3.67 μm for feed rates 0.05 mm/rev, 0.075 mm/rev, and 0.1 mm/rev, respectively.

The results of the above analyses are summarized in Table 8. As listed in Table 8, if minimization of cutting force and surface roughness are equally prioritized, then the following cutting conditions must be used: coolant flow rate of 8 L/min (high coolant flowrate), nose radius of 0.8 mm (high nose radius), cutting speed of 60 m/min (moderate cutting speed), depth of cut of 0.1 mm (small depth of cut), and feed rate of 0.05 mm/rev (low feed rate). If the surface roughness is prioritized, then the following cutting conditions must be used: any coolant flow rates, nose radius of 0.8 mm (high nose radius), cutting speed of 60 m/min (moderate cutting speed), depth of cut of 0.1 mm (small depth of cut), and feed rate of 0.05 mm/rev (low feed rate).

## 4. Concluding Remarks

The concluding remarks of this study are as follows:

- Gamma-TiAl alloys fabricated by Electron Beam Melting (an additive manufacturing process) can replace Ti6Al4V and other similar alloys in high-end aerospace and biomedical applications.
- Since additively fabricated objects made of Gamma-TiAl alloys exhibit poor surface finish, they must be post-processed by traditional manufacturing processes. In this study, turning is utilized as a postprocessing method for EBM-produced Gamma-TiAl. It can be mentioned here that the minimum surface roughness *R_a_* = 0.18 was achieved for the input parameter set of nose radius = 0.8 mm, cutting speed = 80 m/min, depth of cut = 0.1 mm, feed rate = 0.05 mm/rev, and coolant flow rate = 2 L/min. This *R_a_* value is within the applicable level for medical implants [43].
- While postprocessing additively manufactured specimens made of Gamma-TiAl alloys using turning, the following optimal cutting conditions can be used: coolant flow rate of 8 L/min (high coolant flowrate), nose radius of 0.8 mm (high nose radius), cutting speed of 60 m/min (moderate cutting speed), depth of cut of 0.1 mm (small depth of cut), and feed rate of 0.05 mm/rev (low feed rate).
- The above optimal cutting conditions were found by analyzing the experimental data. The experiments were conducted based on Taguchi’s L36 design of experiment, and the data were analyzed using a possibility–probability transformation method. Theis method induces a triangular fuzzy number (possibility distribution) from a given numerical dataset.
- Identifying the optimal cutting conditions requires less computational effort if the abovementioned possibility distribution-based method is used.

## Figures and Tables

**Table 1:** EBM parameters employed for specimen fabrication.

EBM Parameters	Values	Units
Average powder size	110	µm
Acceleration voltage	60	kV
Beam current	18	mA
Beam scanning speed	2200	mm/s
Beam focus offset	0.20	mm

**Table 2:** Physical properties of γ-TiAl.

Properties	Values	Units	Ref.
Ultimate tensile strength	500–630	MPa	[23]
Percentage elongation	0.3–2.5	%	[40]
Hardness	300 ± 30	HV	[23]
Density	3800	kg/m^3^	[41]

**Table 3:** Machining parameters and their selected levels.

Input Parameters	Abbreviations	Symbols	Level 1	Level 2	Level 3
Insert radius (mm)	*r* *_ε_*	A	0.4	-	0.8
Cutting speed (m/min)	*v_c_*	B	40	60	80
Depth of cut (mm)	*a_p_*	C	0.1	0.2	0.3
Feed rate (mm/rev)	*f*	D	0.05	0.075	0.1
Coolant flowrate (L/min)	*Q*	E	2	-	9

**Table 4:** Levels of machining parameters based on L36 standard orthogonal array.

Machining Parameters (Refer to Table 3)						Machining Parameters (Refer to Table 3)					
Exp. No.	A	B	C	D	E	Exp. No.	A	B	C	D	E
1	1	1	1	1	1	19	2	1	2	1	1
2	1	2	2	2	1	20	2	2	3	2	1
3	1	3	3	3	1	21	2	3	1	3	1
4	1	1	1	3	1	22	2	1	2	2	1
5	1	2	2	1	1	23	2	2	3	3	1
6	1	3	3	2	1	24	2	3	1	1	1
7	1	1	1	2	1	25	2	1	3	2	1
8	1	2	2	3	1	26	2	2	1	3	1
9	1	3	3	1	1	27	2	3	2	1	1
10	1	1	1	3	2	28	2	1	3	2	2
11	1	2	2	1	2	29	2	2	1	3	2
12	1	3	3	2	2	30	2	3	2	1	2
13	1	1	2	3	2	31	2	1	3	3	2
14	1	2	3	1	2	32	2	2	1	1	2
15	1	3	1	2	2	33	2	3	2	2	2
16	1	1	2	3	2	34	2	1	3	1	2
17	1	2	3	2	2	35	2	2	1	2	2
18	1	3	1	2	2	36	2	3	2	3	2

**Table 5:** Experimental results regarding cutting force (*F_c_*).

Experiment Numbers	Cutting Conditions					Cutting Force (N)	
	A	B	C	D	E	Trial-1	Trial-2
1	0.4	40	0.1	0.1	2	29.42	28
2	0.4	60	0.2	0.05	2	58.31	54.4
3	0.4	80	0.3	0.075	2	165.62	149.92
4	0.4	40	0.1	0.05	2	28.12	26.39
5	0.4	60	0.2	0.075	2	62.4	52.54
6	0.4	80	0.3	0.1	2	158.77	165.69
7	0.4	40	0.1	0.075	2	33.68	39.12
8	0.4	60	0.2	0.1	2	64.16	67.27
9	0.4	80	0.3	0.05	2	62.71	76.34
10	0.4	40	0.1	0.1	9	43.5	43.95
11	0.4	60	0.2	0.05	9	45.5	50.5
12	0.4	80	0.3	0.075	9	79.19	74.44
13	0.4	40	0.2	0.1	9	67.39	66.29
14	0.4	60	0.3	0.05	9	65.17	72.24
15	0.4	80	0.1	0.075	9	36.44	31.25
16	0.4	40	0.2	0.1	9	68.96	69.34
17	0.4	60	0.3	0.05	9	59.99	60.97
18	0.4	80	0.1	0.075	9	49.62	42.08
19	0.8	40	0.2	0.05	2	55.3	57.58
20	0.8	60	0.3	0.075	2	85.54	90.46
21	0.8	80	0.1	0.1	2	41.69	47.17
22	0.8	40	0.2	0.075	2	65.44	60.6
23	0.8	60	0.3	0.1	2	101.01	103.62
24	0.8	80	0.1	0.05	2	38.7	38.8
25	0.8	40	0.3	0.075	2	88.26	88.06
26	0.8	60	0.1	0.1	2	56.74	51.53
27	0.8	80	0.2	0.05	2	57.23	58.22
28	0.8	40	0.3	0.075	9	83.17	80.18
29	0.8	60	0.1	0.1	9	30.22	44.56
30	0.8	80	0.2	0.05	9	65.42	71.74
31	0.8	40	0.3	0.05	9	75.98	73.19
32	0.8	60	0.1	0.075	9	44.06	49.69
33	0.8	80	0.2	0.1	9	74.2	81.7
34	0.8	40	0.3	0.1	9	96.11	102.67
35	0.8	60	0.1	0.05	9	35.7	36.32
36	0.8	80	0.2	0.075	9	70.19	70.78

**Table 6:** Experimental results regarding surface roughness (*R_a_*).

Experiment Numbers	Cutting Conditions					*R_a_* (µm)					
	A	B	C	D	E	Trial-1			Trial-2		
						Readings					
						1-1	1-2	1-3	2-1	2-2	2-3
1	0.4	40	0.1	0.1	2	0.26	0.26	0.28	0.26	0.32	0.24
2	0.4	60	0.2	0.05	2	0.32	0.34	0.28	0.36	0.34	0.46
3	0.4	80	0.3	0.075	2	0.6	0.62	0.74	0.56	0.88	0.72
4	0.4	40	0.1	0.05	2	0.22	0.24	0.24	0.26	0.26	0.26
5	0.4	60	0.2	0.075	2	0.36	0.36	0.36	0.3	0.32	0.36
6	0.4	80	0.3	0.1	2	0.6	0.68	0.46	0.58	0.58	0.56
8	0.4	60	0.2	0.1	2	0.56	0.56	0.64	0.6	0.56	0.56
9	0.4	80	0.3	0.05	2	0.28	0.3	0.28	0.22	0.24	0.24
10	0.4	40	0.1	0.1	9	0.5	0.5	0.5	0.48	0.48	0.48
11	0.4	60	0.2	0.05	9	0.32	0.36	0.3	0.24	0.24	0.26
12	0.4	80	0.3	0.075	9	0.38	0.4	0.44	0.36	0.4	0.34
13	0.4	40	0.2	0.1	9	0.72	0.72	0.78	0.7	0.72	0.72
14	0.4	60	0.3	0.05	9	0.26	0.3	0.28	0.22	0.26	0.26
15	0.4	80	0.1	0.075	9	0.26	0.26	0.26	0.26	0.28	0.28
16	0.4	40	0.2	0.1	9	0.7	0.62	0.72	0.64	0.52	0.58
17	0.4	60	0.3	0.05	9	0.26	0.32	0.24	0.26	0.26	0.26
18	0.4	80	0.1	0.075	9	0.44	0.46	0.48	0.52	0.46	0.46
19	0.8	40	0.2	0.05	2	0.22	0.22	0.24	0.28	0.24	0.26
20	0.8	60	0.3	0.075	2	0.32	0.38	0.36	0.3	0.3	0.32
21	0.8	80	0.1	0.1	2	0.32	0.32	0.32	0.34	0.34	0.32
22	0.8	40	0.2	0.075	2	0.3	0.26	0.28	0.42	0.32	0.34
23	0.8	60	0.3	0.1	2	0.36	0.4	0.4	0.32	0.34	0.36
24	0.8	80	0.1	0.05	2	0.26	0.24	0.2	0.18	0.18	0.18
25	0.8	40	0.3	0.075	2	0.26	0.22	0.22	0.36	0.3	0.26
26	0.8	60	0.1	0.1	2	0.38	0.34	0.34	0.38	0.4	0.38
27	0.8	80	0.2	0.05	2	0.18	0.22	0.2	0.18	0.24	0.2
28	0.8	40	0.3	0.075	9	0.28	0.4	0.28	0.28	0.38	0.36
29	0.8	60	0.1	0.1	9	0.22	0.26	0.26	0.24	0.24	0.26
30	0.8	80	0.2	0.05	9	0.22	0.22	0.2	0.26	0.22	0.24
31	0.8	40	0.3	0.05	9	0.2	0.22	0.3	0.24	0.24	0.26
32	0.8	60	0.1	0.075	9	0.26	0.2	0.22	0.22	0.2	0.24
33	0.8	80	0.2	0.1	9	0.48	0.48	0.46	0.4	0.4	0.42
34	0.8	40	0.3	0.1	9	0.46	0.48	0.48	0.44	0.48	0.48
35	0.8	60	0.1	0.05	9	0.3	0.3	0.26	0.3	0.28	0.28
36	0.8	80	0.2	0.075	9	0.26	0.28	0.36	0.28	0.34	0.26

**Table 7:** Experimental results regarding surface roughness (*Rt*).

Experiment Numbers	Cutting Conditions					*R_t_* (µm)					
	A	B	C	D	E	Trial-1			Trial-2		
						Readings					
						1-1	1-2	1-3	2-1	2-2	2-3
1	0.4	40	0.1	0.1	2	1.9	2.1	2.2	1.9	2.3	2.5
2	0.4	60	0.2	0.05	2	2.7	2.5	1.8	2.7	2.7	2.8
3	0.4	80	0.3	0.	2	4.6	4.4	4.4	3.7	6	4.9
4	0.4	40	0.1	0.05	2	2.1	1.7	1.8	1.3	1.6	1.7
5	0.4	60	0.2	0.075	2	2.4	2.3	2.1	2	3	5.4
6	0.4	80	0.3	0.1	2	3.5	4.7	3.6	6.8	6.3	5.3
7	0.4	40	0.1	0.075	2	1.6	1.9	2.4	2.6	1.8	2.8
8	0.4	60	0.2	0.1	2	3	3.2	4.2	3.9	2.9	3.1
9	0.4	80	0.3	0.05	2	2	2.5	2.2	1.8	1.6	2.1
10	0.4	40	0.1	0.1	9	2.8	3.5	3.9	2.8	2.6	3.2
11	0.4	60	0.2	0.05	9	2.7	3.2	2.1	2.2	1.9	2.3
12	0.4	80	0.3	0.075	9	6.1	2.8	3.5	2.6	2.8	3.2
13	0.4	40	0.2	0.1	9	3.7	3.7	4.9	3.5	4.1	3.7
14	0.4	60	0.3	0.05	9	2.7	3	2.1	2	2.1	2
15	0.4	80	0.1	0.075	9	2.1	2.2	2.1	1.8	2.4	2.3
16	0.4	40	0.2	0.1	9	4.1	3.5	4.1	4.5	3.2	3.9
17	0.4	60	0.3	0.05	9	4.4	2.9	3.5	3.3	2.5	3.5
18	0.4	80	0.1	0.075	9	2.8	3.3	3.1	3.3	2.7	3.1
19	0.8	40	0.2	0.05	2	1.7	1.8	2	2.5	2	2
20	0.8	60	0.3	0.075	2	2.3	2.3	2.3	1.9	2	2.1
21	0.8	80	0.1	0.1	2	2	1.9	2.5	1.9	1.9	2
22	0.8	40	0.2	0.075	2	2.5	2.1	2.1	4.1	2.9	3.6
23	0.8	60	0.3	0.1	2	2.6	3.2	3.1	2.2	2.7	2.6
24	0.8	80	0.1	0.05	2	1.8	1.6	1.4	2.3	1.2	1.6
25	0.8	40	0.3	0.075	2	6	2.6	1.7	3.3	2.3	2.3
26	0.8	60	0.1	0.1	2	2.8	2.1	2.2	2.4	2.3	2.5
27	0.8	80	0.2	0.05	2	1.3	1.5	1.4	1.2	1.6	1.3
28	0.8	40	0.3	0.075	9	2	3.5	2.4	2.8	2.9	3.6
29	0.8	60	0.1	0.1	9	1.7	1.8	2.4	1.8	2	2.1
30	0.8	80	0.2	0.05	9	2.4	1.9	1.7	1.8	3.4	1.5
31	0.8	40	0.3	0.05	9	4.2	2.2	2	2.1	1.9	3
32	0.8	60	0.1	0.075	9	2	1.9	1.7	1.5	1.6	1.8
33	0.8	80	0.2	0.1	9	3.7	2.5	2.4	2.5	2.6	2.7
34	0.8	40	0.3	0.1	9	3.3	3.6	2.9	2.6	2.7	2.9
35	0.8	60	0.1	0.05	9	2.8	2.6	1.8	4.7	2.2	2.6
36	0.8	80	0.2	0.075	9	1.8	2.2	4	5	3	2.6

**Table 8:** Optimal cutting conditions.

Performance Parameters	Cutting Conditions				
	Coolant Flow Rate (L/min)	Nose Radius (mm)	Cutting Speed (m/min)	Depth of Cut (mm)	Feed Rate (mm/rev)
Cutting force	8	0.8	40, 60	0.1	0.05
*R_a_*	-		60		
*R_t_*	-		60, 80		

## Data Availability

The data presented in this study are available on request from the corresponding author.
